# Impact of type 2 diabetes on lower urinary tract symptoms in men: a cohort study

**DOI:** 10.1186/1471-2490-13-12

**Published:** 2013-02-20

**Authors:** Stephen K Van Den Eeden, Assiamira Ferrara, Jun Shan, Steven J Jacobsen, Virginia P Quinn, Reina Haque, Charles P Quesenberry

**Affiliations:** 1 Division of Research, Kaiser Permanente Northern California, 2000 Broadway, Oakland, CA 94612, USA; 2 Research and Evaluation, Kaiser Permanente Southern California, Pasadena, CA, USA

**Keywords:** Lower urinary tract symptoms, Men, Diabetes, Epidemiology, Cohort study

## Abstract

**Background:**

Studies of the impact of type 2 diabetes on the prevalence and incidence of lower urinary tract symptoms (LUTS) among men have provided divergent results. We sought to examine this issue using two large and diverse cohorts.

**Methods:**

This study used questionnaire and clinical data from two large multiethnic cohorts, the California Men’s Health Study (CMHS) and Research Program in Genes, Environment and Health (RPGEH). Diabetes characteristics data were derived from questionnaire and Diabetes Registry data. LUTS were measured using a standardized scale. Socioeconomic and comorbidity data were obtained by self-report.

Multivariable logistic regression analysis was used to examine the association between baseline DM status and prevalence and incidence of LUTS, with adjustment for potential confounding variables.

**Results:**

We found type 2 diabetes to be associated with prevalent LUTS (odds ratio (OR) = 1.32, 95% confidence interval (CI) 1.26, 1.38). The association was stronger among men with type 2 diabetes who were on active pharmaceutical treatment and had it for a longer duration. No association was observed between type 2 diabetes and new onset LUTS.

**Conclusions:**

Type 2 diabetes increases the risk of LUTS.

## Background

Type 2 diabetes mellitus, lower urinary tract symptoms and benign prostatic hyperplasia are all common disorders that affect men as they age. It is well known that diabetes can negatively impact the bladder and is manifested in later stages as diabetic cystopathy [1,2]. It is less clear, however, how diabetes affects the more common lower urinary tract symptoms (LUTS) of the aging male [2,3].

Early studies reported that diabetes was associated with surgery for enlarged prostate or benign prostatic hyperplasia (BPH) [3,4]. As recently reviewed by Sarma and Parsons [3], most studies determined that type 2 diabetes is associated with a 10-200% increase in the risk of LUTS. However, these studies were quite variable in the way they defined LUTS; for example, the definitions frequently included various markers of BPH, such as medical treatment or surgery. In contrast, other studies have not found type 2 diabetes to be associated with LUTS or BPH [5].

The current study sought to examine the association between type 2 diabetes and LUTS using two large cohorts in which all participants completed a questionnaire that included a standardized assessment of LUTS, the American Urological Association Symptom Index (AUASI) [6].

## Methods

Participants of the California Men’s Health Study (CMHS) and male subjects of the Research Program on Genes, Environment and Health (RPGEH) cohorts formed the study population for this study. Both cohorts were recruited from the membership of Kaiser Permanente in California.

Details of the CMHS have been previously published [7]. Briefly, CMHS baseline data were collected between 2001–2002 on 84,170 men, aged 45 to 69 years as of 1/1/ 2001, who were members of Kaiser Permanente Northern or Southern California regions. A second questionnaire that included the AUASI was administered to the CMHS participants in 2007–2008. Details on the study of LUTS within the RPGEH cohort have been previously published [8]. The RPGEH includes a cohort with baseline data obtained in 2007–2008 on 140,139 men who were adult members of Kaiser Permanente Northern California for at least two years prior to the survey. Data were available for 78,273 CMHS and 106,373 RPGEH men after exclusions for prevalent prostate cancer or missing data. For the analysis related to new onset or incident LUTS, only the 63,245 CMHS men who completed the second assessment and did not have prostate cancer at baseline or after follow-up were included. Informed consent was obtained for the CMHS participants through an information sheet that accompanied the survey and voluntary response. Written informed consent was obtained for the RPGEH participants.

Questionnaire data included race/ethnicity, marital status, birthplace, height, weight, diabetes status, comorbidity (e.g., cardiovascular disease, hypertension, hyperlipidemia, etc.), smoking, alcohol use and physical activity. Physical activity was categorized as minimal, moderate or strenuous based on type of activity and frequency, and consistent with recommendations in a NIH consensus statement on physical activity [9]. Body mass index (BMI) was calculated as weight in kilograms/height in meters^2^ and included in the analyses as an indicator variable with three categories (i.e., <25, 25- < 30, 30+).

Data on LUTS were obtained using the standardized American Urological Association Symptom Index (AUASI) [6] with measurement at baseline for both cohorts and at the follow-up assessment for the CMHS. The AUASI is scored on a 0–35 scale based on seven questions. These data were categorized as no or mild (AUASI score of 0–7); moderate (AUASI 8–19); or severe LUTS (AUASI ≥20). For some analyses, the moderate and severe categories were combined. Incident LUTS was examined only among men with no prostate cancer at baseline or in the follow-up period, who did not undergo BPH treatment before baseline and had an AUASI in the no or mild category (i.e., ≤7). Men were classified as having incident LUTS if they met any of the following criteria: AUASI score increased from ≤7 to 8 or more at the second assessment or underwent treatment for BPH. The latter criterion included selected drug use (e.g., α-blockers or 5-α-reductase inhibitors), undergoing surgery (e.g., a transurethral prostatectomy), or other minimally invasive procedures (e.g., transurethral microwave thermotherapy or transurethral needle ablation).

For analyses that examined diabetes characteristics, the analyses was limited to the Kaiser Permanente Northern California (KPNC) subcohort for linkage to the KPNC Diabetes Registry [10-13].

### Statistical analysis

We first calculated the prevalence of LUTS at baseline by dividing the number of ‘cases’ by the appropriate denominator at the baseline and expressed this percentage by age and diabetes status.

Because many conditions potentially associated with LUTS also tend to vary by diabetes status, we analyzed the data using logistic regression models to obtain odds ratios for LUTS associated with type 2 diabetes adjusted for covariates that may confound that association. We combined moderate and severe LUTS in our analyses. Our analysis adjusted for age, race/ethnicity, physical activity, smoking and BMI. Other factors, such as marital status, and alcohol use, were initially considered but there was no evidence that they were confounding the effect estimates and were therefore not included in regression models. Factors that are known to be a consequence of having type 2 diabetes, such as cardiovascular disease and hyperlipidemia, were not included in our primary analyses. In the combined cohort analyses we also included an indicator variable for cohort. Statistical tests of the regression coefficients were based on the likelihood ratio test and Wald 95% confidence intervals were calculated for each odds ratio.

The study was reviewed and approved by the Institutional Review Boards of Kaiser Permanente Northern and Southern California.

## Results

Except for the age distribution, men were similar in both cohorts with regard to demographic, socioeconomic and comorbid diseases (Table 1). A total of 24,586 or 13.3% had a history of type 2 diabetes. Approximately half of the combined cohorts reported moderate or severe LUTS. Overall, about 13% of the combined cohort members reported fair or poor health. On average, the cohorts were reasonably well educated with just over half having at least some college level courses.

**Table 1:** Selected demographic, socioeconomic, lifestyle and medical characteristics of men in the California men's health study and research program in Genes, environment and health Cohorts

	**Cohort**			
	**CMHS**		**RPGEH**	
	**N = 78,273**		**N = 106,373**	
Demographic & Social	N	(%)	N	(%)
Race				
Asian	6026	7.7	11052	10.4
Black/African American	5656	7.2	10751	10.1
Hispanic	10752	13.7	9009	8.5
White	48838	62.4	72232	67.9
Other/Mixed	7001	8.9	3329	3.1
Lower urinary tract symptoms/AUASI score				
0-7	37036	47.3	52424	49.3
8-19	35917	45.9	47215	44.4
≥20	5320	6.8	6734	6.3
Age (in years)				
18-29			4912	4.6
30-39			8435	7.9
40-49	12853	16.4	17655	16.6
50-59	33703	43.1	24691	23.2
60-69	31717	40.5	27046	25.4
70-79			23634	22.2
80-89				
≥90				
Married	60314	77.1	78804	74.1
Education				
High school or less	14097	18.1	17309	17.6
Some college or trade	27047	34.8	27347	27.9
School				
College or more	36683	47.1	53508	54.5
Household income (per year)				
<$60,000	29676	39.4	34003	34.7
$60-99,999	24640	32.7	28392	29
≥$100,000	21029	27.9	35607	36.3
At least one parent foreign born	28007	35.8	38968	36.6
Medical Co-morbidities				
Fair or poor general health	9684	12.4	15251	14.3
Current or past diabetes	9579	12.2	15007	14.1
Current or past hypertension	28502	36.4	33537	31.5
Cardiovascular disease	13097	16.7	12773	12
Erectile dysfunction	22863	29.2	39646	37.3
Health or behavior characteristics				
Smoking status				
Never smoker	32940	43.4	60570	56.9
Former smoker	34768	45.8	36919	34.7
Current smoker	8275	10.9	8884	8.4
ETOH > = 5 drinks/week	6429	8.2	8455	8
Body mass Index (mean, SD)	27.9	4.8	27.5	5
Vegetables or fruit per week				
3-4 times	10450	13.4	21006	19.8
5 or more times	58457	74.7	74201	69.8
Physical activity (moderate or vigorous) per week				
3-4 times	21310	27.2	32861	30.9
5 or more times	13961	17.8	22269	20.9

The prevalence of men with moderate or severe LUTS increased with age in both cohorts and among men with and without type 2 diabetes (Table 2). While the age specific prevalence was slightly higher among the RPGEH men compared to CMHS men with or without type 2 diabetes, the prevalence was higher among men with type 2 diabetes in all age categories in both cohorts. The severity of LUTS was also increased with age.

**Table 2:** Baseline prevalence of diabetes* and LUTS† by age by cohort

	**Age (years) at baseline**											
	**<45**		**45-49**		**50-59**		**60-69**		**70-79**		**Total**	
	**n**	**(%)**	**n**	**(%)**	**n**	**(%)**	**n**	**(%)**	**n**	**(%)**	**n**	**(%)**
CMHS - DM			908	7.1%	3661	10.9%	5010	15.8%			9579	12.2%
CMHS - No DM			11945	92.9%	30042	89.1%	26707	84.2%			68694	87.8%
			12853		33703		31717				78273	
RPGEH - DM	887	5.91	833	8.0%	3099	12.6%	5030	18.6%	5156	21.8%	15005	14.1%
RPGEH - Not DM	19683	21.54	9599	92.0%	21589	87.4%	22013	81.4%	18476	78.2%	91360	85.9%
LUTS score‡	Diabetes											
0-7	514	57.9%	904	51.9%	2923	43.2%	3513	35.0%	1465	28.4%	9319	37.9%
8-19	339	38.2%	741	42.6%	3374	49.9%	5405	53.8%	2988	58.0%	12847	52.3%
≥20	34	3.8%	96	5.5%	463	6.8%	1122	11.2%	703	13.6%	2418	9.8%
LUTS score‡	Non-Diabetes											
0-7	14059	71.4%	13185	61.2%	27075	52.4%	20153	41.4%	5667	30.7%	80139	50.1%
8-19	5369	27.3%	7769	36.1%	22064	42.7%	24430	50.1%	10648	57.6%	70280	43.9%
≥20	255	1.3%	590	2.7%	2492	4.8%	4137	8.5%	2161	11.7%	9635	6.0%

* From baseline questionnaire data; Diabetes = yes response to having been told by physician had diabetes.

† AUASI score of 8 or more.

‡ Both cohort are combined.

Kaiser Permanente California.

In multivariable models (Table 3), men with type 2 diabetes had higher odds of moderate LUTS than men without this condition (OR = 1.32, 95% CI 1.26-1.38). When men with type 2 diabetes were further classified by treatment status, use of oral antihyperglycemia agents or insulin was associated with an increased odds of LUTS than men without type 2 diabetes. Longer duration of type 2 diabetes was associated with an increased odds of LUTS, although men with shorter duration of type 2 diabetes had a higher odds relative to men without type 2 diabetes. These trends were also apparent when analyses were restricted to the men with type 2 diabetes (data not shown).

**Table 3:** Risk of prevalent lower urinary tract symptoms by diabetes characteristics, CMHS and RPGEH Cohorts, Kaiser Permanente

		**LUTS †**		
		OR *	**95% CI**	
Diabetes	No	1.0 (ref)	ref	ref
	Yes	1.32	1.26	1.38
Treatment	Not DM	1.0 (ref)	ref	ref
	None	1.13	0.97	1.32
	Oral medication	1.40	1.29	1.51
	Insulin	1.28	1.10	1.48
Duration	Not DM	1.0 (ref)	ref	ref
	<5 years	1.28	1.18	1.40
	≥5 years	1.38	1.27	1.51

* Adjusted for age, race/ethnicity, physical activity, smoking, and body mass index.

† AUASI score of 8 or more.

Finally, in the CMHS cohort, type 2 diabetes at baseline questionnaire was associated with a 7% increased odds of LUTS progression (OR = 1.07, 95% CI 0.95, 1.02), as determined by increasing AUASI score or having received treatment for BPH/LUTS after baseline (Table 4). Again, the confidence interval included the null and therefore the data are consistent with no association between DM and LUTS incidence. We did not see an increase in the odds of LUTS with markers of disease severity such as medication use or duration.

**Table 4:** Risk of new onset lower urinary tract symptoms by diabetes characteristics, CMHS Cohort, Kaiser Permanente

		**LUTS †**		
		OR *	**95% CI**	
Diabetes	No	1.0 (ref)	ref	ref
	Yes	1.07	0.95	1.2
Treatment‡	Not DM	1.0 (ref)	ref	ref
	None	1.17	1.03	1.34
	Oral medication	0.87	0.69	1.09
	Insulin	0.91	0.61	1.3
Duration‡	Not DM	1.0 (ref)	ref	ref
	<5 years	1.07	0.92	1.25
	≥5 years	1.05	0.88	1.25

* Adjusted for age, race/ethnicity, physical activity, smoking, and body mass index.

† AUASI score of 8 or more.

‡ CMHS cohort only.

## Discussion

We found clear associations between type 2 diabetes and prevalent lower urinary tract symptoms. Men with type 2 diabetes reported higher AUASI scores in each age group in both cohorts studied. Men with type 2 diabetes were 32% more likely to report LUTS compared to men without type 2 diabetes. The association was stronger with indicators of poorer type 2 diabetes status (e.g., more intensive medical management or duration). In contrast, we found that type 2 diabetes had little if any impact on the risk of developing new LUTS.

The earliest studies of diabetes and LUTS found an association between diabetes and surgery for BPH [4,14,15]. However, surgery for BPH as an endpoint represents a pathway that includes severity of LUTS, the presence of comorbidities that represent surgical contraindications, healthcare access and other concerns. More recent studies have used a mixed definition of BPH that included BPH surgery, symptoms or results of a digital rectal exam [16-19]. These studies have all found associations that consistently point to diabetes affecting voiding function. However, they have not clearly sorted out the underlying mechanism – i.e., dysfunction due to an increase in obstruction secondary to BPH or bladder dysfunction secondary to microvascular and neuropathic effects related to diabetes. However, the evidence of diabetes effect on obstruction is mixed and primarily limited to examination of prostate volume. Sarma et al. [20] found no association between diabetes and prostate volume. Interestingly, they reported a stronger association for irritative LUTS compared to obstructive LUTS. Also relevant to this discussion, Burke et al. [21], using the Olmsted County Study (OCS) data, reported that diabetes was associated with the progression of LUTS, but was not associated with an increase in prostate volume or PSA level. In contrast to our study, Sarma et al. [22], using the same OCS population and the Flint Men’s Health Study, found no association between diabetes medication treatment and progression of LUTS. They did report that the association between diabetes and LUTS seemed to be stronger for irritative symptoms compared to obstructive symptoms. However, the study was limited in that only 101 of the men had diabetes in their analysis. However, other, smaller studies, suggest that diabetes may be related to prostate growth [23]. While another larger study reported fasting glucose to be associated with increased prostate volume, there was an irregular dose–response pattern across quartiles of fasting glucose which limits the interpretation of those data [18]. Nonetheless, these data taken together suggest a bigger impact of diabetes on voiding symptoms unrelated to obstruction, such as the bladder.

It may be that diabetes also adversely affects voiding in combination with other health issues. Kupelian et al. reported an association between metabolic syndrome and LUTS [24]. However, data from the Third National Health and Nutrition Examination Survey (NHANES III) did not find diabetes or most markers of glucose metabolism (or metabolic syndrome) associated with selected lower urinary tract symptoms; unfortunately NHANES did not include a standardized assessment of LUTS [5]. In this study, however, glycosylated hemoglobin was associated with increased prevalence of lower urinary tract symptoms. A study from Austria also failed to find metabolic syndrome (as well as fasting glucose) to be associated with LUTS [25]. In a follow-up of men with type 1 diabetes who were participants in the DCCT/EDIC study, no association between glycosylated hemoglobin (or other markers of disease severity) and LUTS was observed [26].

The relationship between diabetes and progression of LUTS is complicated by possibly multiple changes over time. If diabetes does not increase the prostate volume it may result in a leveling off LUTS in men where obstruction is contributing. However, if diabetes impacts the bladder through vascular and neuropathic mechanisms, it may increase LUTS. Our data on progression do not point to a clear explanation as to which of these mechanisms are operating. The lack of an association with progression in our data may be due to issues related to measurement of progression or a relatively short follow-up.

## Conclusions

We found a clear association between diabetes and prevalent LUTS but no association with new onset LUTS.

## Abbreviations

LUTS: Lower urinary tract symptoms; BPH: Benign prostatic hyperplasia; AUASI: American Urological Association Symptom Index; CMHS: California Men’s Health Study; RPGEH: Research Program on Genes, Environment and Health; BMI: Body mass index; KPNC: Kaiser Permanente Northern California.

## Competing interests

Stephen Van Den Eeden has received salary support from research grants from the National Institutes of Health, GlaxoSmithKline and Takada for studies unrelated to this paper. Assiamira Ferrara has received salary support or research grants from the National Institutes of Health, GlaxoSmithKline, sanofi-aventis and Takada for studies unrelated to this paper. Steven Jacobsen has received research support from the National Institutes of Health and Merck for studies unrelated to this paper. Charles Quesenberry has received salary support or research grants from the National Institutes of Health, GlaxoSmithKline, sanofi-aventis and Takada for studies unrelated to this paper. Jun Shan declares there are no competing interests. Virginia Quinn declares there are no competing interests. Reina Haque declares there are no competing interests.

## Authors’ contributions

SKV designed, helped acquire the data, conducted the analysis, drafted the manuscript and gave final approval to be published. AF helped acquire the data, critically revised the manuscript and gave final approval to be published. JS conducted the analysis, critically revised the manuscript and gave final approval to be published. SJJ critically revised the manuscript and gave final approval to be published. VQ helped acquire the data, critically revised the manuscript and gave final approval to be published. RH critically revised the manuscript and gave final approval to be published. CPQ helped acquire the data, conduct the analysis, critically revised the manuscript and gave final approval to be published. All authors read and approved the final manuscript.

## Authors’ information

Dr. Van Den Eeden is a Research Scientist at the Division of Research at Kaiser Permanente Northern California, a Professor in the Department of Urology at UCSF and a Lecturer in Epidemiology at Stanford University.

## Pre-publication history

The pre-publication history for this paper can be accessed here:

http://www.biomedcentral.com/1471-2490/13/12/prepub
